# Effect of rechallenge nivolumab in a hemodialysis patient with multiple metastases from a rapidly progressed T1a renal clear cell carcinoma: An autopsy case

**DOI:** 10.1002/iju5.12699

**Published:** 2024-02-14

**Authors:** Kazushi Hanawa, Norifumi Sawada, Yuka Yokota, Junki Aikawa, Yuko Otake, Koki Sugimura, Hiroshi Shimura, Takanori Mochizuki, Satoru Kira, Takahiko Mitsui

**Affiliations:** ^1^ Department of Urology, Interdisciplinary Graduate School of Medicine University of Yamanashi Chuo City Yamanashi Japan; ^2^ Department of Human Pathology, Interdisciplinary Graduate School of Medicine University of Yamanashi Chuo City Yamanashi Japan

**Keywords:** autopsy, hemodialysis, metastasis, nivolumab, T1a renal cell carcinoma

## Abstract

**Introduction:**

Distant metastasis of T1a renal cell carcinoma is rare and whether metastasis is more probable in patients undergoing hemodialysis remains unclear. We report the autopsy case of a patient undergoing hemodialysis with multiple metastases that rapidly progressed from T1a renal cell carcinoma treated with multimodal therapy including nivolumab.

**Case presentation:**

A 70‐year‐old male who underwent hemodialysis was diagnosed with clear cell carcinoma (pT1a, G2) after nephrectomy. Six months post‐surgery, bone and lung metastases appeared and treated with radiotherapy and pazopanib, respectively. Nivolumab was administered as second‐ and fourth‐line treatments for lung metastases. The patient died approximately 60 months after initial diagnosis; however, nivolumab controlled disease progression for 24 months. An autopsy revealed the lung's occupation with clear cell carcinoma tumor tissue.

**Conclusion:**

Nivolumab has potential to control lung metastasis progression. Additionally, rechallenge is possible in patients with renal cell carcinoma undergoing hemodialysis.

**Keynote message**

We report the case of a patient undergoing hemodialysis with rapidly progressing T1a RCC controlled with nivolumab for 24 months and autopsy findings.

**Abbreviations & Acronyms**
AE: adverse events
CT: computed tomography
CTCAE: common terminology criteria for adverse events
ESRD: end stage renal disease
HD: hemodialysis
ICI: immune checkpoint inhibitors
irAE: immune‐related adverse event
mRCC: metastasis renal cell carcinoma
OS: overall survival
RCC: renal cell carcinoma
TKI: tyrosine kinase inhibitor

## Introduction

HD patients are at high risk of cancer, and RCC is more common in these patients than in the general population.^1^ The risk of T1a RCC metastasis is generally low^2^; however, there are several reports of aggressive variants that rapidly progress.^3^ Currently, there is no standard treatment for patients with ESRD and metastatic RCC.^4^ Herein, we present the autopsy case of a patient with ESRD and rapidly progressing T1a RCC treated with multimodal therapy, including ICI and radiation therapy.

## Case presentation

**Fig. 1:**
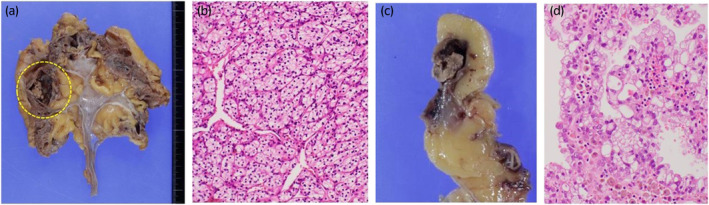
Left kidney specimen (a), clear cell carcinoma, Grade 2 in Fuhrman classification, pT1a. (b) Right kidney specimen (c) and the ACD related RCC, the tumor cells with abundant cytoplasm and round to oval nuclei with prominent nuclei (d).

**Fig. 2:**
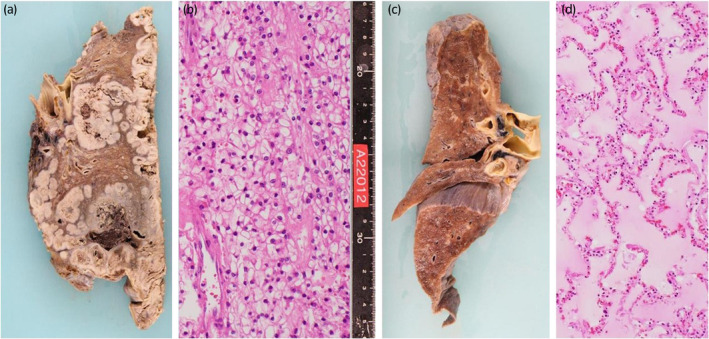
Left lung covered with metastatic lesions. (a) The tumors are all composed of clear cell carcinoma. (b) Right lung showed effusion and no cancer lesion (c). The effusion of the right lung was confirmed microscopically (d).

**Fig. 3:**
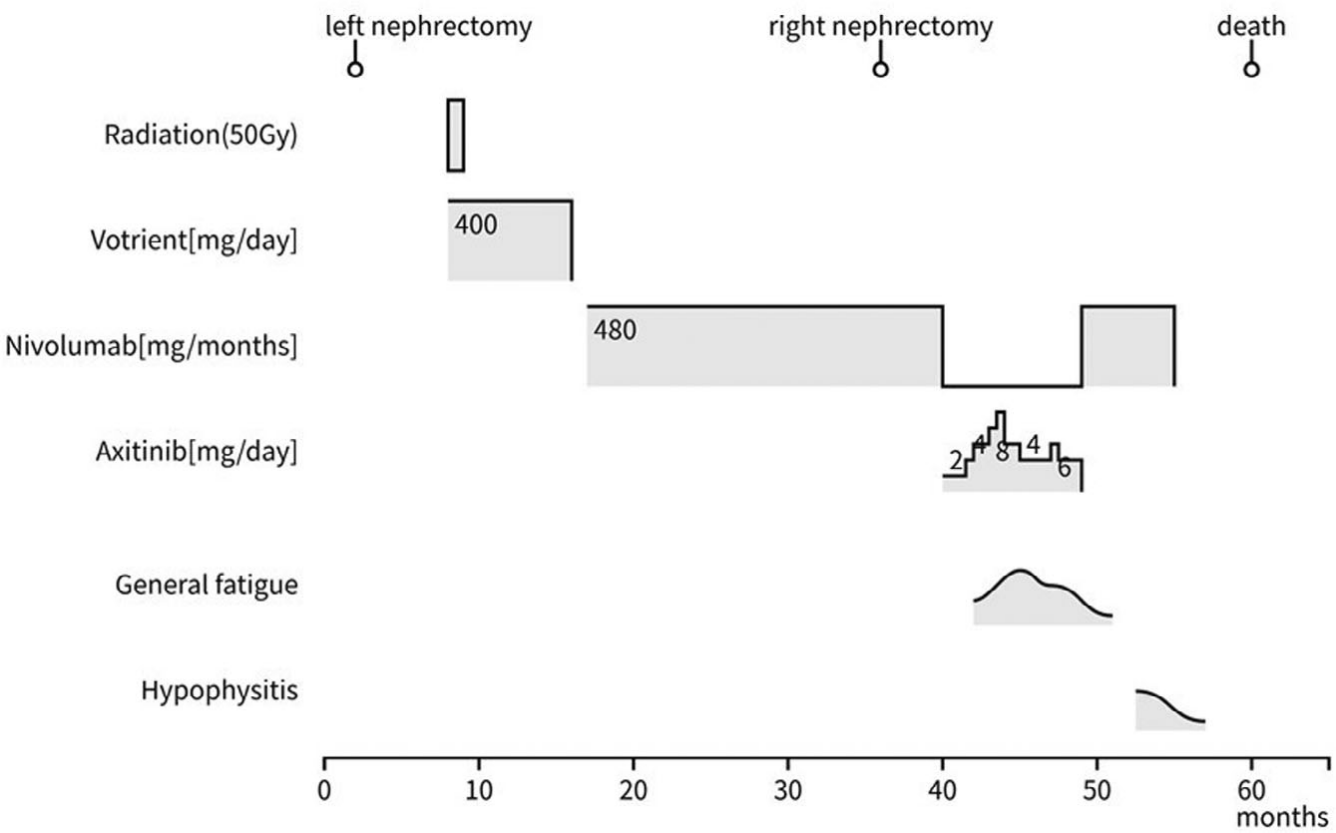
The entire clinical course of the patient. Nivolumab has controlled the disease progression for 24 months.

**Fig. 4:**
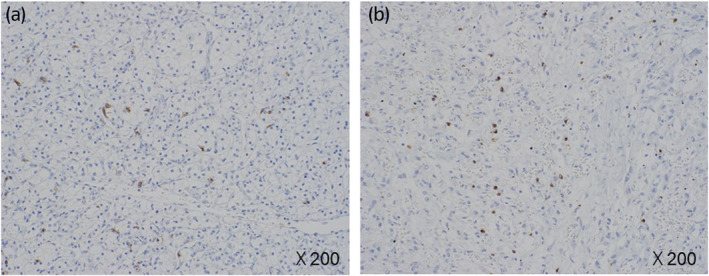
The CD8 staining of primary (a) and metastatic (b) lesion of clear cell RCC were shown. Both lesions showed scarce staining of CD8.

A 70‐year‐old male who had been undergoing HD for 10 years for diabetic nephropathy was referred to our department with a left renal tumor. CT showed a 33 × 37 mm hypervascular tumor in the upper pole of the left kidney. The patient was diagnosed with RCC and underwent laparoscopic left nephrectomy. The pathological diagnosis was clear cell carcinoma, the histological grade was 2 according to the Fuhrman classification, pT1a, and the resection margins were negative (Fig. 1a,b). His Karnofsky Performance Status score was 90. Six months after, right pubic sciatic metastasis and multiple lung metastases were detected using CT. The patient was diagnosed with metastatic RCC of intermediate risk group using the International Metastatic RCC Database Consortium risk classification and treatment of pazopanib (400 mg/day) was started. Radiotherapy at a total dose of 50 Gy was administered to the pubic bone metastases. After 6 months of pazopanib treatment, CT revealed increased lung metastases. Nivolumab (480 mg/4 weeks) was started as second‐line therapy and was effective in maintaining the shrunken size of the lung metastases for 17 months. The right renal tumor mass was diagnosed as RCC, and the patient underwent right laparoscopic nephrectomy. The pathological diagnosis was acquired cystic disease associated RCC. The histological grade was 3, pT1a, and the resection margins were negative (Fig. 1c,d). Six months after, an enlarged left subclavian lymph node appeared, and axitinib was initiated as third‐line therapy. Axitinib was effective for 9 months; however, multiple lung metastases progressed and nivolumab was restarted as fourth line therapy, which retained the metastases size for 6 months. Since the patient's clinical status was stable and not detected any progression on CT, we diagnosed that the best response of this fourth line nivolumab was SD. Hypophysitis was observed during treatment with fourth line nivolumab; however, nivolumab treatment was carefully continued along with hydrocortisone (10 mg/day) supplementation. Lung metastases, new pleural seeding and massive pleural effusions were observed after 6 months of the fourth line nivolumab treatment. Thoracentesis, thoracic drainage, and pleurodesis were then performed. The patient and his family decided to provide the best supportive care instead of further medication. He spent 4 months at home on HD, visited a local hospital three times a week, died of respiratory failure due to multiple lung metastases. After obtaining consent from the patient's family, a post mortem examination was performed. The left thoracic cavity was highly adherent, and grayish‐white nodules that completely occupied the tumor tissue were observed. The right lung contained effusion. Autopsy findings of the metastases were all clear cell carcinomas, suggesting that the metastases originated from the initial clear cell carcinoma (Fig. 2). In the present case, the patient was safely treated with nivolumab for 24 months without CTCAE ver.5 grade 3 or higher AE and treatment showed efficacy (Fig. 3). The staining of CD8 has been performed in the primary and metastatic lesions, and it showed that both of the lesions had scarce staining of CD8 (Fig. 4).

## Discussion

The risk of metastasis is generally low for T1a RCC^2^; however, this case showed metastasis 6 months after the initial left nephrectomy. In a previous study focusing on the comparison between patients with metastatic T1a RCC, and non‐metastatic patients, significant risk factors for metastasis included: symptomatic, C‐reactive protein ≥0.4 mg/dL, grade 3 histological atypia, sarcomatoid component, microvascular invasion and tumor size.^3^ A history of HD was not mentioned in the study and metastatic risk was unknown. RCC's prognosis in patients undergoing HD is better than or equal to that in the general population,^1^ probably because routine screening tests allow for early diagnosis and therapeutic intervention. In a study on the association between clinical symptoms and prognosis in patients with RCC undergoing HD for >6 months, OS and cancer‐specific survival were poor in the symptomatic group. Multivariate analysis showed that a longer HD duration was an independent predictor of prognosis.^5^ Because these studies suggest that long‐term dialysis could be a metastatic risk factor for T1a RCC, it is important to elucidate the therapeutic arm in this population.

Nivolumab is usually used after TKI treatment.^6^ In general, patients undergoing HD are not included in nivolumab's clinical trials, therefore there is little information on the drug's safety and efficacy. A clinical study investigating its safety and efficacy in metastatic patients with RCC and ESRD reported that progression free survival, OS, and the frequency of irAEs were not significantly different between the ESRD and non‐ESRD groups.^7^ Five cases of nivolumab use in patients undergoing HD have been reported^4^, ^8^, ^9^, ^10^ showing good efficacy. During nivolumab treatment, the patient's disease no longer progressed for 24 months. Nivolumab was administered as a rechallenge ICI therapy, which has only recently been reported as a treatment option.^11^ There is also a conflicting report that no significant difference in progression‐free survival was observed between patients treated with the dual regimen and those receiving cabozantinib alone.^12^ Therefore, the treatment of mRCC should be carefully selected. Another finding in this case was the scarce staining of CD8 in the primary and metastatic lesion. Across solid tumors, infiltration by CD8^+^ T cells is associated with an improved prognosis, but paradoxically in clear cell RCC, such infiltration has been associated with worse prognosis.^13^ The reason is reported to be the presence of exhausted CD8^+^ T cells. The scarce staining of CD8 might reflect the longer effect of nivolumab in this case. Nivolumab can be used without volume adjustment because it is degraded into peptides and amino acids. Because of its high molecular weight, it cannot be removed by dialysis. In terms of pharmacokinetics, there are reports of TKI's high efficacy and safety in patients with metastatic RCC undergoing HD.^14^ Thus nivolumab is possible treatment candidate for patients with metastatic RCC undergoing HD and rechallenge can also be adopted.

## Author contributions

Kazushi Hanawa: Conceptualization; data curation; formal analysis; resources; validation; writing – original draft. Norifumi Sawada: Conceptualization; data curation; formal analysis; investigation; methodology; project administration; supervision; validation; writing – review and editing. Yuka Yokota: Methodology; resources; validation; visualization; writing – review and editing. Junki Aikawa: Data curation; resources; supervision; validation. Yuko Otake: Data curation; resources; supervision; validation. Koki Sugimura: Writing – review and editing. Hiroshi Shimura: Writing – review and editing. Takanori Mochizuki: Supervision; validation; visualization; writing – review and editing. Satoru Kira: Supervision; validation; visualization; writing – review and editing. Takahiko Mitsui: Supervision; writing – review and editing.

## Conflict of interest

The authors declare no conflicts of interest.

## Approval of the research protocol by an Institutional Reviewer Board

The protocol for this research project has been approved by University of Yamanashi reviewer board Approval No. 2616.

## Informed consent

Not applicable.

## Registry and Registration No. of the study/trial

Not applicable.

## Compliance with ethical standards

All procedures performed in this study involving human participants were in accordance with the ethical standards of the institutional and/or national research committee and with the 1964 Helsinki declaration and its later amendments or comparable ethical standards.

## References

[1] Tsuzuki T , Iwata H , Murase Y , Takahara T , Ohashi A . Renal tumors in end‐stage renal disease: a comprehensive review. Int. J. Urol. 2018; 25: 780–786. 30066367 10.1111/iju.13759

[2] Sanchez A , Feldman AS , Hakimi AA . Current management of small renal masses, including patient selection, renal tumor biopsy, active surveillance, and thermal ablation. J. Clin. Oncol. 2018; 36: 3591–3600. 30372390 10.1200/JCO.2018.79.2341 PMC6804853

[3] Takayama T , Sugiyama T , Kai F *et al*. Characteristics of aggressive variants in T1a renal cell carcinoma. J. Cancer Res. Clin. Oncol. 2011; 137: 1653–1659. 21874513 10.1007/s00432-011-1040-y PMC11828175

[4] Carlo MI , Feldman DR . Response to nivolumab in a patient with metastatic clear cell renal cell carcinoma and end‐stage renal disease on dialysis. Eur. Urol. 2016; 70: 1082–1083. 27311362 10.1016/j.eururo.2016.05.040 PMC6615055

[5] Hashimoto Y , Takagi T , Kondo T *et al*. Comparison of prognosis between patients with renal cell carcinoma on hemodialysis and those with renal cell carcinoma in the general population. Int. J. Clin. Oncol. 2015; 20: 1035–1041. 25762166 10.1007/s10147-015-0812-9

[6] Escudier B , Porta C , Schmidinger M *et al*. Renal cell carcinoma: ESMO clinical practice guidelines for diagnosis, treatment and follow‐updagger. Ann. Oncol. 2019; 30: 706–720. 30788497 10.1093/annonc/mdz056

[7] Tachibana H , Kondo T , Ishihara H , Takagi T , Tanabe K . Safety and efficacy of nivolumab in patients with metastatic renal cell carcinoma and end‐stage renal disease at 2 centers. Clin. Genitourin. Cancer 2019; 17: e772–e778. 31101580 10.1016/j.clgc.2019.04.004

[8] Ansari J , Ali M , Farrag A , Ali AM , Alhamad A . Efficacy of nivolumab in a patient with metastatic renal cell carcinoma and end‐stage renal disease on dialysis: case report and literature review. Case Rep. Immunol. 2018; 2018: 1623957. 10.1155/2018/1623957 PMC6020478 30009063

[9] Osman‐Garcia I , Congregado‐Ruiz CB , Lendinez‐Cano G , Baena‐Villamarin C , Conde‐Sanchez JM , Medina‐Lopez RA . Outcomes and safety of biweekly and monthly nivolumab in patients with metastatic renal cell carcinoma and dialysis: three case reports and literature review. Urol. Int. 2020; 104: 323–326. 31914452 10.1159/000504515

[10] Shetty AV , Matrana MR , Atkinson BJ , Flaherty AL , Jonasch E , Tannir NM . Outcomes of patients with metastatic renal cell carcinoma and end‐stage renal disease receiving dialysis and targeted therapies: a single institution experience. Clin. Genitourin. Cancer 2014; 12: 348–353. 24565697 10.1016/j.clgc.2014.01.004 PMC4160412

[11] Ravi P , Mantia C , Su C *et al*. Evaluation of the safety and efficacy of immunotherapy rechallenge in patients with renal cell carcinoma. JAMA Oncol. 2020; 6: 1606–1610. 32469396 10.1001/jamaoncol.2020.2169 PMC7260689

[12] Pal SK , Albiges L , Tomczak P *et al*. Atezolizumab plus cabozantinib versus cabozantinib monotherapy for patients with renal cell carcinoma after progression with previous immune checkpoint inhibitor treatment (CONTACT‐03): a multicentre, randomised, open‐label, phase 3 trial. Lancet 2023; 402: 185–195. 37290461 10.1016/S0140-6736(23)00922-4 PMC11017728

[13] Fridman WH , Pages F , Sautes‐Fridman C , Galon J . The immune contexture in human tumours: impact on clinical outcome. Nat. Rev. Cancer 2012; 12: 298–306. 22419253 10.1038/nrc3245

[14] Czarnecka AM , Kawecki M , Lian F , Korniluk J , Szczylik C . Feasibility, efficacy and safety of tyrosine kinase inhibitor treatment in hemodialyzed patients with renal cell cancer: 10 years of experience. Future Oncol. 2015; 11: 2267–2282. 26260806 10.2217/fon.15.112

